# Supplementary material for: Exploring Sea Lice Vaccines against Early Stages of Infestation in Atlantic Salmon (Salmo salar)
Source: Vaccines (Basel). 2022 Jul 1;10(7):1063. doi: 10.3390/vaccines10071063 (PMC9324576; doi:10.3390/vaccines10071063)

Supplementary figure

Figure S1. Experimental design of vaccination trial. A: Experimental groups were used in the trial. B: Temporary work line and sample analysis methods. Each circle represents a tank.

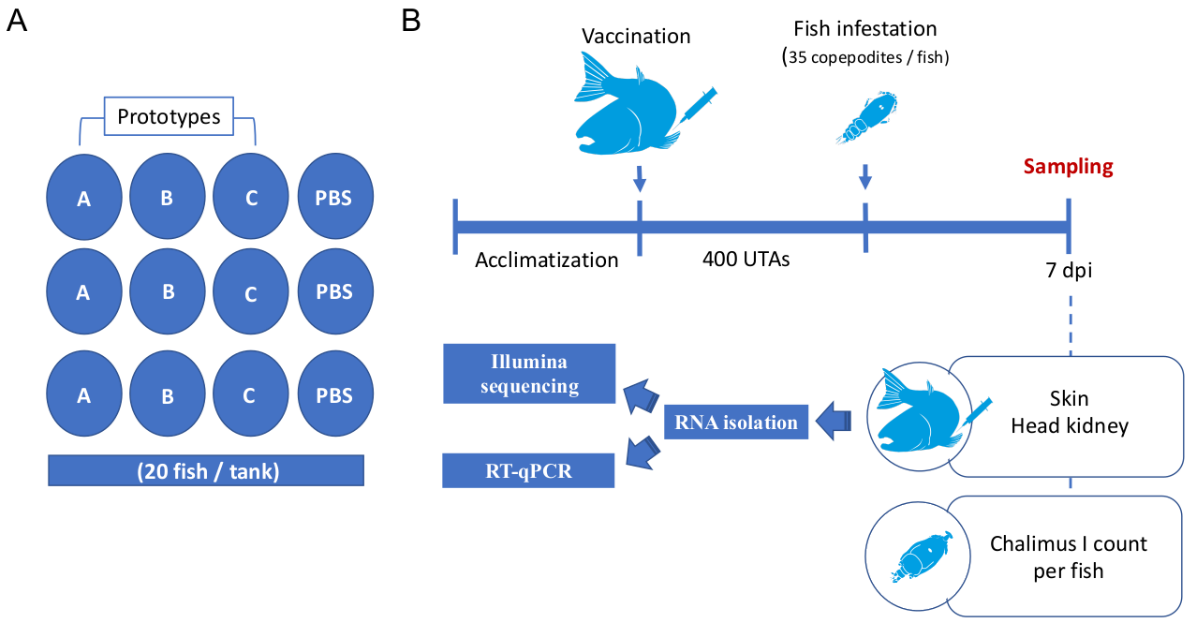

Supplement: Supplementary file 1 [file vaccines-10-01063-s001.zip › Supplementary figure.pdf]
