# Supplementary material for: Exploring Sea Lice Vaccines against Early Stages of Infestation in Atlantic Salmon (Salmo salar)
Source: Vaccines (Basel). 2022 Jul 1;10(7):1063. doi: 10.3390/vaccines10071063 (PMC9324576; doi:10.3390/vaccines10071063)
Supplement: Supplementary file 1 [file vaccines-10-01063-s001.zip › Supplementary TableS1.pdf]

Table S1. Primers list used for RT-qPCR analysis.

Primer Name	Gene	Sequence 5'-3'	Tm	Efficiency (%)	Reference
EFα_F	*Elongation factor-(reference gene)*	TGCTGGTGGTGTGGGTGAGT	60	95.18	[41]
EFα_R		CCTCAAACCGCTTCTGGCTGT			
IgM_F	*Inmunoglobulin M*	TGAGGAGAACTGTGGGCTACACT	60	99,98	[38]
IgM_R		TGTTAATGACCACTGAATGTGCAT			
IgM_Prove		CATCAGATGCAGGTCC			
IgT_F	*Inmunoglobulin T*	CAACACTGACTGGAACAACAAGGT	60	107,77	[38]
IgT_R		CGTCAGCGGTTCTGTTTTGGA			
IgT_Prove		AGTACAGCTGTGTGGTGCA			
COX-2_F	*Cyclooxygenase 2*	CAGTGCTCCCAGATGCCAAG	60	102,34	[41]
COX-2_R		GCGAAGAAGGCGAACATGAG			
IL1B_F6	*Interleukin-1B*	GATCTGGAGGTATCCCATCA	60	122,36	[41]
IL1B_R6		CACAGCACTCTCCAGCAAGA			
MHCII_F	*Major histocompatibility complex II*	CTCCTCAAAGGACCTGCAGG	60	104,72	[41]
MHCII_R		TCAGGACCTTTGTTCCAGGC			
TLR22_3_F	*Toll-like recePtor 22*	TGCCTTCTAACCCTCTCCCT	61	91,36	[41]
TLR22_3_R		CGCTGCTCTCAGACAGGAAG			
FerritinM_F1	*Ferritin M*	TATCACCACGATTGCGAAGC	60	102,66	[41]
FerritinM_R1		CTCGTCGCTGTTCTCCTTGA			
GSHPx_F1	*Glutathione peroxidase*	TAAAGTGGTGCTGATCGAGA	54	100,23	[41]
GSHPx_R1		GTTCTCCTGATGTCCGAACT			
Blv r_F1	*Biliverdin reductase*	AAACAGATCCACCAGCCAGG	59	106,7	[41]
Blv r_R1		ACAGCCGACTTTAAGCAGCT			
Hep_F1	*Hepcidin*	GCTGTTCCTTTCTCCGAGGTGC	59	111,07	[41]
Hep_R1		GTGACAGCAGTTGCAGCACCA			
HO_F2	*Heme oxygenase*	GTCCTCTCGAGTGGTGAAGC	61	94,21	[41]
HO_R2		ATCTCTGAGTCCCTGGCCAA			
ALA s_F1	*Aminolevulinate synthase*	GGTAGGATGCCTGCTGACTG	63	105,52	[41]
ALA s_R1		CCCCAAGCCTGTTTTGCTGA			
ALA d_F2	*Aminolevulinate dehydratase*	CCACTCGCCCATCCATCATA	59	126,72	[41]
ALA d_R2		ACACCTCACATGGACACTGT			
SOD_F1	*Superoxide dismutase*	CCGTATTCTTTGAGCAGGAG	54	104,98	[41]
SOD_R1		AGCCGTTGGTGTTGTCTC			
